# Effectiveness of sacituzumab govitecan in metastatic triple-negative breast cancer: a real-world retrospective cohort study from Central Europe

**DOI:** 10.1093/oncolo/oyag014

**Published:** 2026-01-23

**Authors:** Małgorzata Pieniążek, Anna Polakiewicz-Gilowska, Justyna Żubrowska, Lenka Rušinová, Hana Študentová, Aleksandra Konieczna, Agnieszka Młodzińska, Karolina Winsko-Szczęsnowicz, Maja Lisik-Habib, Anika Pękala, Daniel Krejčí, Jan Šustr, Iveta Kolářová, Iwona Danielewicz, Magdalena Szymanik-Resko, Renata Soumarová, Tomasz Ciszewski, Miloš Holánek, Bogumiła Czartoryska-Arłukowicz, Aleksandra Łacko, Jolanta Smok-Kalwat, Michał Jarząb, Renata Pacholczak-Madej, Miroslava Malejčíková, Zuzana Bielčiková, Mirosława Püsküllüoğlu

**Affiliations:** Department of Oncology, Wroclaw Medical University, Wrocław 50-367, Poland; Lower Silesian Comprehensive Cancer Center, Wrocław, 53-413 Poland; Breast Cancer Unit, Maria Skłodowska-Curie National Research Institute of Oncology, Gliwice Branch, Gliwice 44-102, Poland; Department of Clinical Oncology, Holy Cross Cancer Center, Kielce 25-734, Poland; Department of Oncology, Stefan Kukura Hospital Michalovce, Michalovce 071 01, Slovakia; Department of Oncology, Faculty of Medicine and Dentistry, Palacky University and University Hospital, Olomouc 779 00, Czech Republic; Department of Breast Cancer and Reconstructive Surgery, Maria Sklodowska-Curie National Research Institute of Oncology, Warsaw 02-781, Poland; Department of Breast Cancer and Reconstructive Surgery, Maria Sklodowska-Curie National Research Institute of Oncology, Warsaw 02-781, Poland; Department of Clinical Oncology, M. Sklodowska-Curie Bialystok Oncology Center, Białystok 15-027, Poland; Department of Proliferative Diseases, Nicolaus Copernicus Multidisciplinary Centre for Oncology and Traumatology, Łódź 93-513, Poland; Department of Proliferative Diseases, Nicolaus Copernicus Multidisciplinary Centre for Oncology and Traumatology, Łódź 93-513, Poland; Department of Oncology, First Faculty of Medicine, Charles University in Prague and Bulovka University Hospital, Prague 121 08, Czech Republic; Department of Oncology and Radiotherapy, Faculty of Medicine in Pilsen, Charles University and University Hospital Pilsen, Plzen 301 00, Czech Republic; Department of Oncology and Radiotherapy, Faculty of Medicine in Hradec Kralove and University Hospital in Hradec Kralove, Charles University, Hradec Kralove 500 05, Czech Republic; Department of Clinical Oncology, Maritime Hospital in Gdynia, Gdynia 81-519, Poland; Department of Clinical Oncology, Maritime Hospital in Gdynia, Gdynia 81-519, Poland; Department of Oncology, Third Faculty of Medicine, Charles University, University Hospital Kralovske Vinohrady, Prague 128 44, Czech Republic; Department of Metabolic Diseases and Immuno-Oncology, Medical University of Lublin, Lublin 20-400, Poland; Department of Comprehensive Cancer Care, Masaryk Memorial Cancer Institute and Faculty of Medicine, Masaryk University, Brno 656 53, Czech Republic; Department of Clinical Oncology, M. Sklodowska-Curie Bialystok Oncology Center, Białystok 15-027, Poland; Department of Oncology, Wroclaw Medical University, Wrocław 50-367, Poland; Lower Silesian Comprehensive Cancer Center, Wrocław, 53-413 Poland; Department of Clinical Oncology, Holy Cross Cancer Center, Kielce 25-734, Poland; Breast Cancer Unit, Maria Skłodowska-Curie National Research Institute of Oncology, Gliwice Branch, Gliwice 44-102, Poland; Department of Gynecological Oncology, Maria Sklodowska-Curie National Research Institute of Oncology, Krakow Branch, Krakow 31‐115, Poland; Department of Anatomy, Jagiellonian University Medical College, Kraków 31-008, Poland; II Oncology Clinic of LFUK and NOÚ, National Cancer Institute, Bratislava 833 10, Slovakia; Department of Oncology, First Faculty of Medicine, Charles University and General University Hospital, Prague 121 08, Czech Republic; Department of Clinical Oncology, Maria Sklodowska-Curie National Research Institute of Oncology, Krakow Branch, Krakow 31-115, Poland

**Keywords:** metastatic triple-negative breast cancer, antibody–drug conjugate, sacituzumab govitecan, real-world data, progression-free survival, safety

## Abstract

**Background:**

Sacituzumab govitecan (SG), an antibody–drug conjugate targeting TROP-2, demonstrated superior efficacy over standard chemotherapy in heavily pretreated metastatic triple-negative breast cancer (mTNBC) in the ASCENT trial. However, real-world data remain limited. This study evaluated the effectiveness and safety of SG in an unselected multinational cohort of patients with mTNBC.

**Methods:**

This retrospective analysis included 303 women who initiated SG treatment between August 2021 and April 2025 across 18 oncology centers in Poland, the Czech Republic and Slovakia, within the Central European Breast Cancer Collaboration (CEBCC-102). Primary endpoints were median progression-free survival (mPFS) and overall survival (mOS). Secondary objectives included response pattern, safety and identification of factors influencing outcomes. Adverse events (AEs) were graded using Common Terminology Criteria for AE, version 5.0, and treatment response was assessed according to Response Evaluation Criteria in Solid Tumors (RECIST), version 1.1.

**Results:**

The median follow-up was 8.8 months (IQR 4.5-14.4). The mPFS was 4.4 months, and mOS was 11.3 months. The overall response rate was 30.7%. The most frequent AEs were hematologic: neutropenia (69.0%) and anemia (39.6%). In multivariate Cox analyses, poor ECOG performance status and liver metastases were independently associated with worse outcomes. Diarrhea and hypersensitivity reactions showed favorable prognostic associations.

**Conclusions:**

In this largest real-world cohort, the clinical benefit of SG observed in the ASCENT trial was confirmed under routine practice conditions. Poor performance status and liver metastases predicted inferior outcomes, while certain treatment-related AEs warrant further investigation.

Implications for Practice This large real-world study confirms that sacituzumab govitecan provides meaningful clinical benefit for patients with metastatic triple-negative breast cancer outside the controlled setting of clinical trials. Treatment outcomes were consistent with the pivotal ASCENT trial, supporting the reliability of this therapy in everyday practice. Greater benefit was observed in individuals with good performance status and without liver metastases. The occurrence of selected treatment-related adverse events, such as diarrhea or hypersensitivity reactions, may be associated with improved outcomes, highlighting the need for further research on markers of treatment response.

## Introduction

Triple-negative breast cancer (TNBC) represents the least common subtype of breast cancer, yet it is frequently associated with a highly aggressive clinical course.^1^^,^^2^ Patients are typically diagnosed at a younger age. The disease is characterized by early relapse, high metastatic potential and poor overall outcomes.^2^ Although genomic profiling has revealed that TNBC is a biologically heterogeneous entity, in routine clinical practice it continues to be defined in simplified terms as the absence of estrogen receptor (ER), progesterone receptor (PR), and human epidermal growth factor receptor 2 (HER2) overexpression by immunohistochemistry (IHC) or HER2 gene amplification by molecular testing.^3^^,^^4^ Despite the identification of molecular subtypes, targeted therapies directed at specific alterations have so far provided limited benefit and a significant unmet clinical need remains, particularly in patients with advanced disease.^3^

For many years, systemic chemotherapy was the only available standard of care in metastatic TNBC (mTNBC) until the landmark ASCENT trial established a new therapeutic option.^5^ This pivotal study demonstrated that sacituzumab govitecan (SG), a trophoblast cell surface antigen 2 (TROP2) directed antibody–drug conjugate (ADC), significantly prolonged both progression-free survival (PFS) and overall survival (OS) compared with standard chemotherapy in patients with mTNBC previously exposed to at least 2 lines of therapy.^5^ More recently, emerging data have also indicated superiority of SG in combination with pembrolizumab over chemotherapy plus pembrolizumab in the first-line treatment of programmed death-ligand 1 (PD-L1)-positive (combined positive score [CPS] ≥10) mTNBC.^6^ In the phase III ASCENT-03 trial of patients previously untreated, advanced TNBC ineligible for PD-1/PD-L1 inhibitors, SG significantly improved mPFS compared with chemotherapy, with a similar safety profile and longer duration of response.^7^ SG consists of a humanized monoclonal antibody targeting TROP-2 linked to the active metabolite of irinotecan (SN-38), allowing targeted delivery of a potent topoisomerase I inhibitor to tumor cells and a clinically relevant bystander effect.^8^^,^^9^

While clinical trials have firmly established the efficacy of SG, real-world studies are essential to complement these findings, as they encompass broader and more diverse patient populations and provide critical insights for clinical decision-making. Real-world data largely confirm SG benefit observed in the ASCENT trial, although some reports describe slightly shorter median PFS and OS, as well as lower overall response rate (ORR), likely reflecting the more heavily pretreated and heterogeneous populations encountered in routine practice.^5^^,^^10–16^ Moreover, real-world studies offer valuable insights into the role of clinical biomarkers during SG treatment, thereby allowing for a more comprehensive contextualization of therapeutic outcomes in daily clinical settings.^17^^,^^18^

The present multicenter cohort study within Central European Breast Cancer Collaboration (CEBCC-102) aimed to evaluate the real-world effectiveness and safety of SG in patients with mTNBC.

## Methods

### Study population and treatment

Between August 2021 and April 2025, a total of 303 women initiated treatment with SG therapy across 18 departments of clinical oncology in Poland, the Czech Republic, and Slovakia (listed in Table S1—Supplementary Appendix 1). Patient identification was performed through hospital registry systems. Eligibility criteria across 3 countries were largely consistent and reflected the ASCENT trial protocol.^5^ Common requirements included histologically confirmed TNBC, inoperable or metastatic disease.^19–21^ SG could be administered if the patient had previously received 2 or more systemic therapies, including at least one for advanced disease. Treatment was continued until disease progression, unacceptable toxicity or death, whichever occurred first, or discontinued based on physician or patient decision. The detailed reimbursement criteria in each country are provided in Supplementary Appendix 1. Patients enrolled in early-access reimbursement programs outside clinical trials were also included. Discrepancies were adjudicated by a senior author of the study.

**Table 1.** Characteristic of patients and initial treatment.

Parameter		Total (*N* = 303)
**Age at SG therapy start (years)**	Mean (SD)	54.5 (12.26)
	Median (quartiles)	53.87 (45.33-65.03)
	Range	26.63-86.06
	*n*	303
**Relevant comorbidities**	No	166 (54.8%)
	Yes	137 (45.2%)
**Number of relevant chronic conditions**	Mean (SD)	0.84 (1.21)
	Median (quartiles)	0 (0-1)
	Range	0-6
	*n*	303
**Menopausal status at SG initiation**	Pre-menopause	113 (37.3%)
	Post-menopause	190 (62.7%)
**BMI (kg/m²)**	Mean (SD)	25.96 (4.95)
	Median (quartiles)	25 (22.62-28.74)
	Range	15.81-53.33
	*n*	303
**BMI categories**	Normal weight	144 (47.5%)
	Underweight	8 (2.6%)
	Overweight	91 (30.0%)
	Obesity	60 (19.8%)
**PS at SG initiation**	PS 0	125 (41.3%)
	PS 1	166 (54.8%)
	PS 2	10 (3.3%)
	PS 3	1 (0.3%)
	Unknown	1 (0.3%)
**Initial treatment**	Palliative	62 (20.5%)
	Curative	241 (79.5%)
**Time from NPL diagnosis to SG therapy (years)**	Mean (SD)	1.29 (1.32)
	Median (quartiles)	0.84 (0.48-1.7)
	Range	0-8.43
	*n*	300
**Initial staging**	Locoregional	245 (80.9%)
	Metastatic	58 (19.1%)
**Radical surgery performed**	No	59 (19.5%)
	Yes	232 (76.6%)
	Unknown	12 (4%)
**Radical radiotherapy performed**	No	96 (31.7%)
	Yes	193 (63.7%)
	Unknown	14 (4.6%)
**Previous palliative systemic treatment lines**	Mean (SD)	1.76 (1.13)
	Median (quartiles)	1 (1-2)
	Range	1-10
	*n*	303

Abbreviations: BMI, body mass index; NPL, neoplasm; PS, performance status; SG, sacituzumab govitecan.

The starting dose of SG was 10.0 mg/kg, unless modified by the treating physician. Adverse events (AEs) were collected and graded using the National Cancer Institute Common Terminology Criteria for AE (NCI-CTCAE), version 5.0.^22^ AEs were captured from routine visits and laboratory reports. Dose reductions or treatment interruptions in response to AEs were implemented according to the Summary of Product Characteristics for the European Union.^23^ Treatment response was assessed using computed tomography or magnetic resonance imaging according to Response Evaluation Criteria in Solid Tumors (RECIST), version 1.1,^24^ every 8 weeks in Poland and in accordance with the local standards in the Czech Republic and Slovakia. Radiological response evaluations categorized responses into complete response (CR), partial response (PR), stable disease (SD), or progressive disease (PD). The objective tumor response rate (ORR) was calculated based on the proportion of patients achieving either CR or PR in routine clinical practice. It was assessed at each participating center in accordance with local procedures.

This study was designed and reported in line with the STrengthening the Reporting of OBservational studies in Epidemiology extension for Real-World Evidence (STROBE-RWE) and the European Society of Medical Oncology—Guidance for Reporting Oncology Real-World Evidence (ESMO-GROW) guidance for reporting outcomes in real-world data.^25^^,^^26^

### Data collection

For this retrospective cohort study, data were extracted from both electronic and paper-based medical records and organized into predefined categories to enable a comprehensive analysis. Patient-specific data included age, sex, menopausal status and the number of chronic conditions. Disease-related data encompassed the date of breast cancer diagnosis, HER2 status (IHC and/or fluorescence in situ hybridization [FISH]) from initial or most recent specimens, conversion from hormone receptor (HR) positivity, initial staging (locoregional vs metastatic) and treatment intent at diagnosis (curative vs palliative). The course of curative-intent treatment, including systemic therapy, surgery and radiotherapy, was also recorded. Baseline characteristics at SG initiation included ECOG performance status, height, weight, starting SG dose, reason for treatment initiation, and sites of metastatic disease (lungs, central nervous system [CNS], bones, lymph nodes, liver, skin/subcutaneous tissue, serous fluid or other). The number of measurable lesions according to RECIST 1.1 and size of the biggest lesion were documented. Treatment history captured the number and type of prior palliative systemic therapy lines. SG therapy-related data included treatment dates (first and last), number of cycles and infusions, treatment status (ongoing vs discontinued), and reasons for discontinuation, as well as performance status at the end of SG. Information on dose modifications (delays, reductions, timing and causes) was also collected. Safety data comprised SG-related AEs with details on onset and severity. Hypersensitivity reactions were retrospectively identified from infusion visit notes describing AEs occurring during the infusion or within 30 minutes thereafter. In the database, the term “hypersensitivity reactions” encompassed a broad clinical spectrum, from mild infusion-related symptoms to severe, potentially life-threatening reactions, including anaphylactic shock. Reported events included dyspnea, hypotension, flushing, erythema, chest discomfort, wheezing, edema, urticaria, anaphylaxis, mouth ulceration, skin exfoliation, swollen tongue and throat tightness. Efficacy data encompassed best response assessed by RECIST 1.1, whereas follow-up information included progression details, subsequent therapies, last visit date, survival status and, if applicable, date of death. Data cut-off was on July 1, 2025.

### Primary and secondary objectives

The primary aim of this study was to determine median OS and PFS in an unselected multinational cohort of patients with mTNBC receiving SG. OS was measured from the first dose of SG until death, and PFS from SG treatment initiation until disease progression or death. Secondary objectives explored response to treatment, safety, and identification of factors influencing outcomes.

### Statistical analysis

For each continuous variable, the mean and standard deviation (SD) as well as the median with first and third quartiles (Q1-Q3) and range were reported to provide a comprehensive overview of the data. Categorical variables were presented as absolute numbers and corresponding percentages. The total number of available observations (*n*) was indicated for each parameter when data were incomplete.

Univariate and multiple Cox regression (proportional hazards model) was employed to model the potential impact of predictors on a time to event. HRs (hazard ratios), alongside the 95% CIs, were presented. Variables that showed a significant or near-significant association (*P* < .20) with the dependent variable were included in the multivariate model, based on the authors’ previous analytical experience. Significance level was set to .05.

### Ethical considerations

The study received ethics approval in Poland (Maria Skłodowska-Curie Institute: Warsaw no. 21/2024, 22.02.2024; Kraków no. 2/2023, 18.04.2023) and in the Czech Republic (Masaryk Institute Brno no. 1737/2025, 10.06.2025; General University Hospital Prague protocol 110825 S-IV, 21.08.2025). Procedures followed the Declaration of Helsinki and national laws. Patients consented to SG within reimbursement programs; committees confirmed that no additional consent was needed for this retrospective study.

## Results

### Baseline patient characteristics

Clinical data were collected from 303 women. The median age at treatment initiation was 54 years (IQR: 45-65). The study cohort consisted predominantly of postmenopausal women (62.7%). Relevant comorbidities were present in 45.2% of patients and the majority (49.8%) had a BMI above 25 kg/m^2^. Only 11 patients initiated SG with an ECOG performance status >1. Almost 80% women had previously undergone curative treatment. A comprehensive summary of patient characteristics and initial treatment is provided in Table 1.

TNBC was diagnosed de novo in most patients, while receptor conversion from HR-positive disease was documented in 21.1% of cases, which was notably more frequent than conversion from HER2-positivity, observed in only 2.3%. The most common sites of metastases were lymph nodes (67.7%) and lungs (53.1%). Nearly three-quarters of patients presented with up to 10 metastatic lesions. The biggest size of metastatic lesion on CT performed prior to treatment initiation had a median of 30.0 mm (IQR: 20.0-49.5, range 2-202). Tumor burden of metastatic disease was described in details in Table 2.

**Table 2.** Tumor burden characteristics.

Parameter		Total (*N* = 303)
**Reason for SG treatment initiation**	Metastatic	295 (97.4%)
	Non-operable	8 (2.6%)
**Lung metastases**	No	142 (46.9%)
	Yes	161 (53.1%)
**Liver metastases**	No	204 (67.3%)
	Yes	99 (32.7%)
**CNS metastases**	No	274 (90.4%)
	Yes	28 (9.2%)
	Unknown	1 (0.3%)
**Bones metastases**	No	181 (59.7%)
	Yes	122 (40.3%)
**Lymph nodes (non-regional) metastases**	No	98 (32.3%)
	Yes	205 (67.7%)
**Skin/subcutaneous tissue metastases**	No	210 (69.3%)
	Yes	93 (30.7%)
**Other sites metastases**	No	253 (83.5%)
	Yes	50 (16.5%)
**Fluid**	No	249 (82.1%)
	Yes	54 (17.8%)
**Number of measurable lesions**	None	12 (4%)
	1-3 lesions	110 (36.3%)
	4-10 lesions	117 (38.6%)
	10-20 lesions	7 (2.3%)
	Over 20 lesions	42 (13.9%)
	Unknown	15 (5%)
**Size of the biggest lesion (mm)**	Mean (SD)	39.63 (29.88)
	Median (quartiles)	30 (20-49.5)
	Range	2-202
	*n*	275

Abbreviations: CNS, central nervous system; *N*, group size, SG, sacituzumab govitecan.

### Disease burden according to performance status

Patients with PS 0 and PS 1 showed comparable disease burden with similar frequencies of lung metastases (PS 0: 65 [52.0%] vs PS 1: 87 [52.4%]) and lymph node involvement (84 [67.2%] vs 113 [68.1%]). Median lesion size was identical in both groups (29.5 mm; PS 0: IQR 19.3-46.8, range 4-202 vs PS 1: IQR 20.0-50.0, range 2-170). The proportion of patients with >20 metastatic lesions was also similar (17 [13.6%] vs 22 [13.3%]).

Patients with PS 2-3 (*n* = 11) showed a higher disease burden with more frequent bone metastases (6 [54.5%] vs 40 [32.0%] in PS 0 and 76 [45.8%] in PS 1), a higher proportion of >20 metastatic lesions (3 [27.3%] vs ∼13% in PS 0-1), and larger median lesion size (43 mm; IQR 20-64, range 17-136).

Due to the limited number of patients with PS 2-3, these comparisons should be interpreted with caution.

### The palliative systemic treatment before sacituzumab govitecan

Before the initiation of SG patients had received a median of one (IQR: 1-2) palliative systemic treatment line. The number of previous palliative systemic treatment lines was not associated with OS (HR = 1.06, 95% CI 0.94-1.2; *P* = .4). Similarly, the number of prior palliative treatment lines did not significantly affect PFS, although a numerical trend was observed (HR = 1.11, 95% CI 1.0-1.23; *P* = .06). Most frequently administered agents in previous palliative settings included platinum compounds (53.1%), taxanes (42.6%), and anthracyclines (38.6%). Other commonly used drugs comprised gemcitabine (29.0%), capecitabine (25.1%) and various other cytostatics (26.7%). Chemotherapy with pembrolizumab was reported in 12.5% of patients and PARP inhibitors in 6.3%. Only a minority had received hormonal therapy (7.6%) or anti-HER2 agents (2.0%), which corresponds with receptor conversion.

### Treatment exposure and outcomes

The median follow-up was 8.8 months (IQR 4.52-14.41). At the data cut-off, 254 patients (83.8%) had completed SG treatment, primarily due to disease progression (*n* = 233, 91.7%). Patients completed a median of 6 (IQR: 3-11) full cycles of SG. Treatment delays due to AEs occurred in 191 patients (63.0%), while 114 patients (37.6%) required dose reductions.

Regarding tumor response, SD was observed in 102 patients (33.7%), PR in 87 (28.7%) and CR in 6 (2.0%), yielding an ORR of 30.7%. PD, as the best treatment response, was reported in 83 patients (27.4%), while response data were unavailable for 25 (8.3%).

### Adverse events

The most frequently reported AE was neutropenia, occurring in 69.0% of patients, followed by alopecia (47.5%), anemia (39.6%), and fatigue (38.3%). The rarest AEs were neutropenic fever (4.3%) and hypersensitivity reactions to SG (5.9%). Overall, hematologic toxicities predominated over non-hematologic events in this cohort. Summary of AEs is available in Table 3.

**Table 3.** Adverse events during sacituzumab govitecan treatment.

Side effect	Any grade, *N* (%)	Grades and cases (%) per side effect					
		1	2	3	4	5	Lack of data
**Neutropenia**	209 (69.0)	21 (10.0)	51 (24.4)	90 (43.1)	46 (22.0)	–	1 (0.5)
**Alopecia**	144 (47.5)	Not collected					–
**Anemia**	120 (39.6)	42 (35)	56 (46.7)	17 (14.2)	5 (4,2)	–	–
**Fatigue**	116 (38.3)	41 (35.3)	51 (44)	23 (19,8)	1 (0.9)	–	–
**Nausea**	72 (23.8)	28 (38.9)	34 (47.2)	8 (11.1)	1 (1.4)	–	1 (1.4)
**Diarrhea**	59 (19.5)	24 (40.7)	15 (25.4)	18 (30.5)	2 (3.4)	–	–
**Liver toxicity**	50 (16.5)	26 (52)	14 (28)	9 (18)	–	–	1 (2)
**Vomiting**	32 (10.6)	14 (43.8)	12 (37.5)	6 (18.8)	–	–	–
**Thrombocytopenia**	30 (9.9)	12 (40)	5 (16.7)	10 (33.3)	2 (6.7)	–	1 (3.3)
**Hypersensitivity reactions to SG**	18 (5.9)	7 (38.9)	3 (16.7)	6 (33.3)	2 (11.1)	–	–
**Febrile neutropenia**	13 (4.3)	–	–	5 (38.5)	5 (38.5)	1 (7.7)	2 (15.4)

Abbreviations: *N*, group size; SG, sacituzumab govitecan.

### Survival outcomes

**Figure 1.**
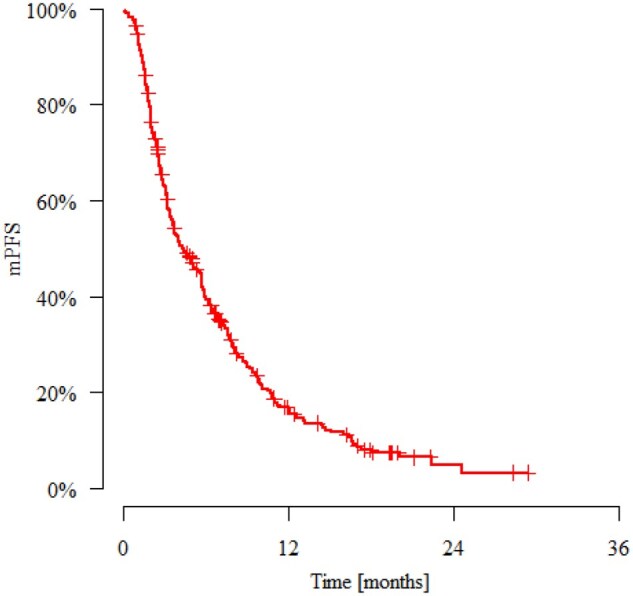
Kaplan–Meier curve of progression-free survival of study population. Abbreviation: mPFS, median progression-free survival.

**Figure 2.**
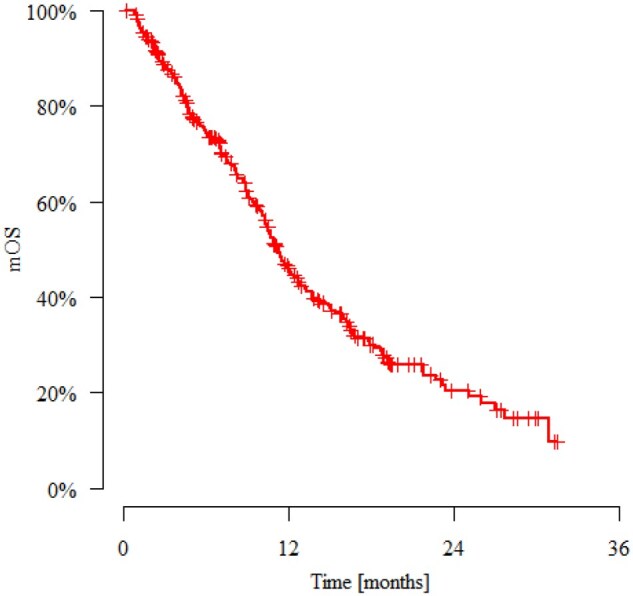
Kaplan–Meier curve of overall survival of study population. Abbreviation: mOS, median overall survival.

The mPFS was 4.37 months. The estimated PFS rates at 3, 6, 9, and 12 months were 62.9%, 39.9%, 26.0%, and 16.3%, respectively (Figure 1). In the overall study population, the mOS was 11.3 months. The estimated survival rates at 3, 6, 9, and 12 months were 88.7%, 75.0%, 62.2%, and 46.0%, respectively (Figure 2).

### Factors influencing progression free survival

In univariate Cox analyses, several baseline and treatment-related factors were significantly associated with PFS. Poor performance status (PS 2-3 vs 0) strongly predicted shorter PFS, as did the presence of lung, liver or combined lung/liver/CNS metastases. Conversely, treatment exposure and certain AEs were linked to improved PFS: each additional completed cycle of SG, as well as the occurrence of diarrhea, hypersensitivity reactions, neutropenia or alopecia, correlated with longer PFS.

In multivariate Cox regression, after adjustment for covariates meeting the prespecified inclusion threshold (*P* < .20), PS 2-3 continued to independently predict poorer PFS. The presence of liver metastases was also an independent adverse prognostic factor. Notably, hypersensitivity reactions to SG emerged as an independent predictor of improved PFS. Other metastasis sites, BMI categories, dose reductions due to AEs and other AEs showed no significant independent association. All variables associated with PFS are summarized in Table 4.

**Table 4.** Factors associated with progression-free survival in univariate and multivariate Cox proportional hazards models.

Variable	Category	Univariate HR (95% CI)	*P*	Multivariate HR (95% CI)	*P*
**PS at SG initiation**	0	Reference		Reference	
	1	0.97 (0.75-1.25)	.80	0.69 (0.5-0.97)	.03*
	2-3	4.715 (2.506-8.870)	<.001*	4.928 (1.939-12.528)	.001*
**Liver metastases**	No	Reference		Reference	
	Yes	1.51 (1.17-1.97)	.002*	1.65 (1.16-2.35)	.005*
**SG dose reduction due to AEs**	Yes vs No	0.69 (0.53-0.89)	.004*	0.806 (0.565-1.150)	.24
**Best response**	SD	Reference		Reference	
	PD	13.78 (8.97-21.18)	<.001*	4.36 (2.62-7.27)	<.001*
	CR	0.913 (0.333-2.503)	.86	0.16 (0.04-0.7)	.02*
**Hypersensitivity reactions to SG**	Yes vs No	0.5 (0.28-0.89)	.02*	0.210 (0.095-0.468)	<.001*
**Diarrhea**	Yes vs No	0.667 (0.477-0.93)	.02*	0.9 (0.59–1.37)	.63
**Neutropenia**	Yes vs No	0.52 (0.4-0.68)	<.001*	1.02 (0.68–1.52)	.94
**Alopecia**	Yes vs No	0.64 (0.5-0.83)	.001*	0.95 (0.7–1.3)	.76

Abbreviations: AE, adverse event; CR, complete response; HR, hazard ratio; PD, progressive disease; PS, performance status; SD, stable disease; SG, sacituzumab govitecan.

* *P* < .05.

### Factors influencing overall survival

In univariate analysis, poorer ECOG performance status, presence of visceral metastases and disease burden were significantly associated with shorter OS. Specifically, PS 1 and PS 2-3 were linked to markedly worse outcomes, while lung, liver, CNS, and bone involvement also predicted shorter OS. A longer interval from metastatic diagnosis to SG initiation was associated with improved OS. PD vs SD was strongly adverse, whereas alopecia and hypersensitivity reactions showed favorable trends.

In multivariate analysis, after adjustment for covariates meeting the prespecified inclusion threshold (*P* < .20), ECOG PS remained the strongest independent predictor of poor OS with PS 2-3 carrying over a tenfold higher risk of death compared with PS 0. Liver metastases also independently predicted shorter OS, while treatment delays due to AEs were associated with longer survival. Diarrhea emerged as an independent favorable factor. The association of neutropenia with improved OS in univariate analysis reversed after adjustment, suggesting confounding by treatment duration and response dynamics. All variables associated with OS are summarized in Table 5.

**Table 5.** Factors associated with overall survival in univariate and multivariate Cox proportional hazards models.

Variable	Univariate HR (95% CI)	*P*-value	Multivariate HR (95% CI)	*P*-value
**ECOG performance status**				
**0**	Reference	–	Reference	–
**1**	1.79 (1.31-2.45)	<.001*	1.58 (1.02-2.47)	.04*
**2–3**	7.93 (3.88-16.22)	<.001*	10.80 (3.51-33.22)	<.001*
**Metastatic sites**				
**Lung metastases**	1.52 (1.13-2.05)	.005*	1.44 (0.99-2.10)	.06
**Liver metastases**	1.85 (1.37-2.50)	<.001*	2.46 (1.61-3.74)	<.001*
**CNS metastases**	1.58 (1.00-2.50)	.05*	0.76 (0.40-1.44)	.4
**Bone metastases**	1.53 (1.15-2.05)	.004*	1.04 (0.69-1.57)	.85
**Interval from metastatic diagnosis to SG initiation (per year)**	0.79 (0.68-0.91)	.001*	0.87 (0.74-1.03)	.1
**Best response**				
**SD**	Reference	–	Reference	–
**PD**	5.47 (3.76-7.95)	<.001*	2.60 (1.48-4.57)	.001*
**SG administration delays due to AEs**	0.61 (0.45-0.82)	.001*	0.50 (0.31-0.82)	.006*
**Adverse events**				
**Diarrhea**	0.75 (0.51-1.11)	.15	0.58 (0.35-0.96)	.04*
**Hypersensitivity reactions**	0.50 (0.24-1.01)	.05	0.47 (0.19-1.15)	.1
**Alopecia**	0.65 (0.48-0.88)	.005*	0.88 (0.58-1.32)	.53
**Neutropenia**	0.57 (0.42-0.78)	<.001*	2.35 (1.41-3.92)	.001*

Abbreviations: AE, adverse event; HR, hazard ratio; PD, progressive disease; PS, performance status; SD, stable disease; SG, sacituzumab govitecan.

* *P* < .05.

## Discussion

In this large real-world cohort of 303 women with metastatic mTNBC treated with SG the mPFS and mOS were 4.4 and 11.3 months, respectively, with an ORR of 30.7%. These results confirm the clinical activity of SG in an unselected, real-world population. Hematologic toxicities (mainly neutropenia in 69.0% cases) predominated over non-hematologic events in this cohort. Poor performance status and liver metastases were the strongest adverse prognostic factors. An intriguing and novel observation from our study is the favorable prognostic association of selected treatment-related AEs—particularly diarrhea and hypersensitivity reactions—with improved survival outcomes.

Diarrhea is a well-recognized, expected toxicity of SG, attributable to the SN-38 payload and has been previously associated with treatment delays, dose interruptions, or discontinuation, potentially compromising drug exposure and efficacy.^27^ However, an alternative explanation for our findings may be that patients experiencing diarrhea received more proactive and intensive supportive care, including early antidiarrheal treatment (consistent with recently published studies) and closer monitoring, allowing continuation of therapy at effective cumulative doses despite toxicity.^28^

Additionally, this association may reflect underlying pharmacogenetic variability. Polymorphisms in UGT1A1, particularly the UGT1A1*28 allele, are known to impair SN-38 glucuronidation, resulting in increased systemic exposure and a higher risk of gastrointestinal toxicity, including diarrhea, as well as neutropenia. Recent real-world data suggest that while UGT1A1*28 homozygosity is associated with increased toxicity-related treatment discontinuation, it may also be indicative of higher drug exposure without compromising antitumor efficacy in patients able to remain on treatment.^27^

A similar exposure–response relationship may also explain the favorable prognostic association observed for hypersensitivity reactions. Hypersensitivity and infusion-related reactions to SG are well characterized and typically occur after repeated dosing, reflecting immune sensitization in the context of ongoing drug exposure rather than idiosyncratic early intolerance.^29^ Importantly, severe hypersensitivity events and anaphylaxis remain rare and only infrequently lead to permanent treatment discontinuation, while the majority of reactions are manageable with standard premedication, close monitoring and supportive measures.^29^ Thus, the occurrence of hypersensitivity reactions in patients able to continue therapy may serve as an indirect marker of prolonged and biologically effective drug exposure. In this context, hypersensitivity—similar to diarrhea—may represent an “on-treatment” phenomenon rather than a detrimental event, aligning with the concept of on-target toxicity as a surrogate of adequate systemic exposure, which has been described for other ADCs.^30–32^

Although pharmacokinetic data and formal immunologic correlates were not available in our dataset, these observations should be regarded as hypothesis-generating. Prospective studies integrating pharmacogenetic profiling, pharmacokinetics and immune biomarkers are warranted to clarify whether selected treatment-related toxicities could inform treatment optimization and patient monitoring strategies.

The survival outcomes are broadly consistent with, though slightly lower than, those observed in the pivotal phase III ASCENT trial (mPFS 5.6 months, mOS 12.1 months, ORR 35%).^5^ The modest difference likely reflects the more heterogeneous and less selected population in routine clinical practice, including patients with ECOG performance status >1, extensive prior therapy, and comorbidities. Importantly, this alignment underscores the robustness of SG efficacy beyond controlled trial settings.

Our results are also in line with previously published real-world data from Europe and the United States. Reported mPFS across real-world data cohort range between 3.9 and 5.2 months, and mOS between 8.6 and 13.1 months.^10–15^ Outcomes in our Polish-Czech-Slovak cohort therefore fall well within the international range. Slightly shorter survival times observed in the French and UK cohorts likely reflect inclusion of more heavily pretreated or clinically fragile patients, while longer OS reported in smaller German studies may be due to more selected populations.^10^^,^^11^^,^^13^ Notably, the most recent Spanish multicenter analysis demonstrated mPFS and mOS virtually identical to our results despite inclusion of patients with ECOG ≥2 and CNS metastases.^16^

The toxicity profile was comparable to the pivotal clinical trial: neutropenia (69% in our analysis vs 63% in ASCENT) and alopecia (47% vs 46%) were the most common AEs, while diarrhea occurred less frequently in our cohort (19% vs 59% in ASCENT).^5^^,^^33^ This discrepancy likely reflects underreported diarrhea in routine practice. Importantly, rates of febrile neutropenia (4.3%) and hypersensitivity (5.9%) remained low, confirming manageable safety with standard supportive care.

Toxicity patterns were also consistent across real-world studies with neutropenia, anemia, diarrhea, fatigue and alopecia representing the most frequent treatment-related AEs.^10^^,^^12–16^ The incidence of neutropenia varied from 32.6% and 59.1% in other real-world data reports to 69% in our cohort, confirming hematologic toxicity as the most prevalent but manageable side effect. Dose reductions due to AEs occurred in about one third to one half of patients across studies, mirroring our 37.6% rate.^10^^,^^12–16^

The findings that SG cycle delays and treatment delays due to AEs were associated with longer survival indirectly support the results of the ASCENT trial, in which patients treated with SG who experienced dose interruptions achieved a higher ORR (39%) compared with those without dose interruptions (29%), suggesting that treatment delays or cycle modifications due to AEs do not diminish—and may even reflect—greater therapeutic benefit.^33^ Importantly, this should be clearly distinguished from upfront dose attenuation. Beyond these findings, real-world evidence also indicates that an initial SG dose reduction of ≥20% is associated with significantly worse treatment outcomes with markedly shorter PFS and OS compared with patients who initiated therapy with <20% dose reduction. This effect persisted in multivariable analysis, where a ≥ 20% initial dose reduction emerged as an independent risk factor for disease progression and death, while no safety benefit was observed, as the incidence of AEs (including grade ≥3) and the use of G-CSF were comparable between groups.^34^

Taken together, these findings support the hypothesis that selected treatment-related AEs may reflect an exposure–response relationship rather than purely detrimental toxicity. While such an association was not confirmed in the post hoc ASCENT analysis,^35^ it warrants further exploration in translational studies integrating pharmacokinetic and biomarker data. Understanding whether these AEs serve as surrogate indicators of adequate drug delivery or systemic exposure could ultimately refine patient monitoring and dose optimization strategies.

Across real-world studies, performance status and hepatic involvement consistently emerge, as the main determinants of prognosis in patients treated with SG. Similar to our findings, in the United States, the French and UK cohorts identified ECOG PS ≥ 2 as the strongest independent predictor of shorter PFS and OS, confirming that baseline functional status remains the key clinical factor influencing outcomes with SG.^11^^,^^13^^,^^36^ Likewise, the adverse prognostic role of liver metastases, observed in our multivariate model, has been uniformly reported in French and Spanish real-world analyses.^13^^,^^16^

In our analysis, treatment response patterns were also strongly prognostic: PD was associated with markedly worse outcomes—both PFS and OS, whereas CR was highly favorable for PFS. These findings likely reflect 2 biologically distinct patterns of disease behavior—one sensitive to therapy and the other highly resistant.^33^^,^^37^

These findings reinforce the transformative role of SG in the management of mTNBC. The reproducibility of outcomes across real-world settings provides strong reassurance of its effectiveness in less selected, more heterogeneous populations. However, several knowledge gaps remain. Predictive biomarkers of response are still lacking and RWD on quality of life, long-term outcomes and treatment sequencing remain limited. The optimal positioning of SG in relation to immunotherapy and other ADCs is an evolving question of high clinical relevance.^38^

Looking ahead, the therapeutic landscape of mTNBC is rapidly evolving. The excellent results observed in PD-L1-positive populations and ASCENT-03 data, showing a highly statistically significant and clinically meaningful improvement in PFS,^7^ underscore the expanding potential of SG beyond later-line settings. Future research should focus on integrating SG earlier in the treatment paradigm, evaluating rational combinations with immunotherapy or targeted agents, and identifying molecular or clinical predictors that can guide individualized treatment selection.

### Study limitations

This study has several important limitations. Its retrospective and observational design inherently limits the ability to establish causal relationships and introduces potential selection bias. The study population was restricted to patients meeting national reimbursement criteria, which resulted in relatively homogeneous baseline characteristics. This may have reduced the capacity to detect additional prognostic factors beyond those already established, although the multinational nature of the cohort strengthens the generalizability of the findings. The key pathological assessments were performed according to local procedures. The lack of centralized review may have contributed to inter-laboratory variability. Similarly, treatment response and toxicity assessments, though based on standardized RECIST 1.1 and CTCAE v5.0 criteria, were conducted by multiple investigators across participating centers, potentially introducing differences related to local practice and experience. The retrospective data collection relied on existing documentation, which might have been incomplete for certain clinical or safety parameters, leading to possible underreporting of AEs. In addition, data on UGT1A1 polymorphisms were not available, precluding any assessment of their potential impact on treatment-related toxicity or systemic exposure. Sociodemographic variables, including socioeconomic status, ethnicity, and regional healthcare access, were not systematically recorded, although they may influence treatment exposure and outcomes. Finally, the median follow-up period was relatively short. Despite these limitations, the study provides valuable multicenter, real-world insights from 3 Central European countries, reflecting routine clinical practice and supporting the external validity of pivotal trial findings in a broader patient population.

## Conclusions

This largest international cohort confirms that the clinical benefit of SG demonstrated in the ASCENT trial is reproducible in real-world oncology practice. Poor performance status and the presence of liver metastases consistently emerged as independent predictors of worse outcomes. An intriguing observation was the association of selected treatment-related AEs with improved outcomes, a finding that warrants further investigation.

## Supplementary Material

oyag014_Supplementary_Data

## Data Availability

Data generated and analyzed in this study are available from the corresponding author upon reasonable request. Supplemental material for this article is available online.
