# Supplementary material for: Effectiveness of sacituzumab govitecan in metastatic triple-negative breast cancer: a real-world retrospective cohort study from Central Europe
Source: Oncologist. 2026 Jan 23;31(3):oyag014. doi: 10.1093/oncolo/oyag014 (PMC12923110; doi:10.1093/oncolo/oyag014)
Supplement: oyag014_Supplementary_Data [file oyag014_supplementary_data.zip › Supplementary_Appendix_saci_CEBCC.docx]

Supplementary Appendix

Methods

**Reimbursement rules in Poland, Czech Republic and Slovakia**

1. **Inclusion and exclusion criteria in Polish drug reimbursment program**

**Eligibility criteria**

Patients must meet all of the following criteria:

1. age over 18 years;
2. histologically confirmed advanced breast cancer, defined as:
   a) metastatic breast cancer (stage IV), or
   b) locally advanced breast cancer (stage III) when radical local treatment (surgery or radiotherapy) is ineffective or not feasible;
3. histologically confirmed triple-negative breast cancer (TNBC);
4. documented lack of steroid hormone receptor expression (estrogen receptors and progesteron receptors <1%);
5. documented lack of HER2 overexpression (IHC 0 or 1+) or HER2 gene amplification (negative by in situ hybridization (ISH));
6. presence of evaluable disease per RECIST 1.1;
7. ECOG performance status of 0–1;
8. exclusion of pregnancy and breastfeeding;
9. no presence of life-threatening visceral metastases;
10. absence of significant comorbidities that contraindicate therapy, as determined by the treating physician based on the relevant Summary of Product Characteristics (SmPC) and national oncology guidelines;
11. no contraindications to the drug;
12. absence of untreated or progressing CNS metastases and no significant neurological symptoms or need to increase corticosteroid dose within 1 month prior to treatment initiation;
13. adequate organ function confirmed by blood laboratory tests, allowing safe initiation of therapy in the treating physician’s opinion;
14. In case of coexisting malignancies or history of other malignancies, patients may be eligible upon approval by the National or Regional Consultant, provided prior treatment was radical or led to complete remission.

All criteria must be fulfilled simultaneously.

Patients previously treated under alternative funding schemes may also be enrolled, provided that they met the above eligibility criteria at treatment initiation.

**Exclusion Criteria**

Patients are excluded from the program in the event of:

1. disease progression;
2. clinically significant deterioration of the patient's condition due to cancer, even without radiographic or clinical evidence of progression;
3. life-threatening treatment toxicity according to current CTCAE criteria;
4. recurrent or unacceptable grade 3–4 treatment-related toxicity (treatment may resume after symptom resolution or reduction to grade 1–2);
5. decline in performance status to ECOG 3–4;
6. hypersensitivity to the drug, murine protein, or any excipient precluding continued treatment;
7. clinically significant decline in quality of life as assessed by the treating physician;
8. pregnancy or breastfeeding, unless risk-benefit assessment by the physician and National or Regional Consultant supports continued therapy.
9. **Reimbursement in Czech Republic according to State Institute for Drug Control – SÚKL (Státní ústav pro kontrolu léčiv)**
   Treatment with sacituzumab govitecan is reimbursed for adult patients with inoperable or metastatic triple-negative breast cancer who have previously received two or more systemic therapies, of which at least one was indicated for advanced disease. Reimbursement continues until disease progression or the occurrence of unacceptable toxicity. The patient must meet all of the following conditions:
   a) ECOG performance status of 0–1;
   b) prior treatment should have included an anthracycline and a taxane, except for patients who were not eligible for such therapy;
   c) no evidence of clinically active brain metastases, or brain metastases are adequately treated.
10. **Reimbursement in Slovakia according to State Institute for Drug Control - ŠÚKL (Štátny ústav pre kontrolu liečiv)**
    Reimbursed treatment is indicated for adult patients with unresectable or metastatic triple-negative breast cancer who have received at least two prior systemic treatment regimens, of which at least one was administered for advanced or metastatic disease, provided that all of the following criteria are met:
    a) prior neoadjuvant or adjuvant therapy for localized disease qualifies as one of the required prior regimens only if unresectable, locally advanced, or metastatic disease developed within 12 months after completion of such therapy,
    b) the patient has an ECOG performance status of 0–1,
    c) prior treatment included a taxane, unless it was contraindicated or deemed inappropriate.

Treatment is reimbursed until disease progression or the occurrence of intolerable toxicity, whichever occurs first, and is subject to prior approval from the health insurance provider.

Table S1: Participating centers

| Center No. | Institution Name | City | Country | Type of Center | Number of Patients |
| --- | --- | --- | --- | --- | --- |
| 1 | Maria Skłodowska-Curie National Research Institute of Oncology, Gliwice Branch | Gliwice | Poland | National Research Institute | 42 |
| 2 | Lower Silesian Comprehensive Cancer Center | Wrocław | Poland | Comprehensive Cancer Center | 36 |
| 3 | Department of Comprehensive Cancer Care, Masaryk Memorial Cancer Institute and Faculty of Medicine, Masaryk University | Brno | Czech Republic | Comprehensive Cancer Center | 34 |
| 4 | Department of Breast Cancer and Reconstructive Surgery, Maria Skłodowska-Curie National Research Institute of Oncology | Warsaw | Poland | National Research Institute | 30 |
| 5 | Nicolaus Copernicus Multidisciplinary Centre for Oncology and Traumatology | Łódź | Poland | Regional Oncology Center | 23 |
| 6 | M. Skłodowska-Curie Bialystok Oncology Center | Białystok | Poland | Regional Oncology Center | 22 |
| 7 | Holy Cross Cancer Center, Department of Clinical Oncology | Kielce | Poland | Regional Oncology Center | 18 |
| 8 | Department of Oncology, First Faculty of Medicine, Charles University and General University Hospital | Prague | Czech Republic | University Hospital | 17 |
| 9 | Department of Oncology and Radiotherapy, Faculty of Medicine in Hradec Kralove and University Hospital in Hradec Kralove, Charles University | Hradec Kralove | Czech Republic | University Hospital | 14 |
| 10 | Maria Skłodowska-Curie National Research Institute of Oncology, Krakow Branch | Kraków | Poland | National Research Institute | 13 |
| 11 | Department of Oncology, First Faculty of Medicine, Charles University and Bulovka University Hospital | Prague | Czech Republic | University Hospital | 12 |
| 12 | Department of Clinical Oncology, Maritime Hospital | Gdynia | Poland | Regional Oncology Center | 11 |
| 13 | Department of Oncology, Third Faculty of Medicine, Charles University, University Hospital Kralovske Vinohrady | Prague | Czech Republic | University Hospital | 10 |
| 14 | II Oncology Clinic of LFUK, National Cancer Institute | Bratislava | Slovakia | National Cancer Institute | 8 |
| 15 | Department of Oncology and Radiotherapy, Faculty of Medicine, Palacký University and University Hospital | Olomouc | Czech Republic | University Hospital | 5 |
| 16 | Department of Oncology, Medical University Clinical Hospital No. 1 | Lublin | Poland | University Hospital | 4 |
| 17 | Department of Oncology and Radiotherapy, Faculty of Medicine in Pilsen, Charles University and University Hospital Pilsen | Plzeň | Czech Republic | University Hospital | 3 |
| 18 | Department of Oncology, Stefan Kukura Hospital | Michalovce | Slovakia | Regional Hospital | 1 |
